# Supplementary figures and images for: A complex genetic interaction implicates that phospholipid asymmetry and phosphate homeostasis regulate Golgi functions
Source: PLoS One. 2020 Jul 30;15(7):e0236520. doi: 10.1371/journal.pone.0236520 (PMC7392219; doi:10.1371/journal.pone.0236520)

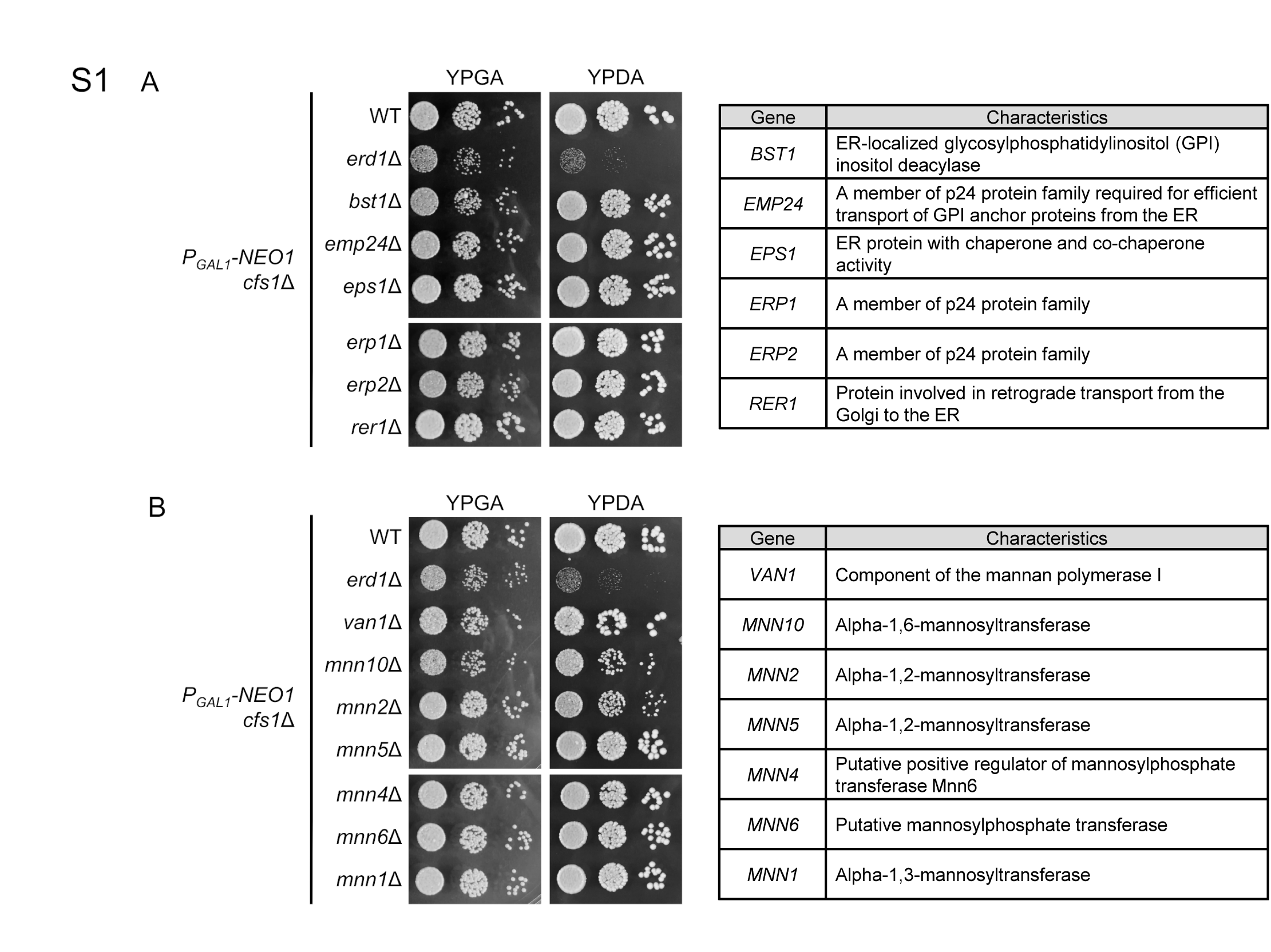

Supplement: S1 Fig — (A) Neo1p-depleted cfs1Δ is not synthetically lethal with other mutations involved in ER retention. Cells were spotted onto YPGA (galactose) and YPDA (glucose) plates and grown as described in Fig 1B. The strains used were PGAL1-NEO1 cfs1Δ (WT) (YKT2085) and PGAL1-NEO1 cfs1Δ carrying erd1Δ (YKT2136), bst1Δ (YKT2206), emp24Δ (YKT2207), eps1Δ (YKT2208), erp1Δ (YKT2209), erp2Δ (YKT2210), and rer1Δ (YKT2211). (B) Neo1p-depleted cfs1Δ is not synthetically lethal with mutations involved in Golgi glycosylation. Cells were spotted onto YPGA (galactose) and YPDA (glucose) plates and grown as described in (A). The strains used were PGAL1-NEO1 cfs1Δ (WT) (YKT2085) and PGAL1-NEO1 cfs1Δ carrying erd1Δ (YKT2136), van1Δ (YKT2212), mnn10Δ (YKT2213), mnn2Δ (YKT2214), mnn5Δ (YKT2215), mnn4Δ (YKT2216), mnn6Δ (YKT2217), and mnn1Δ (YKT2218). (TIF) [file pone.0236520.s001.tif]

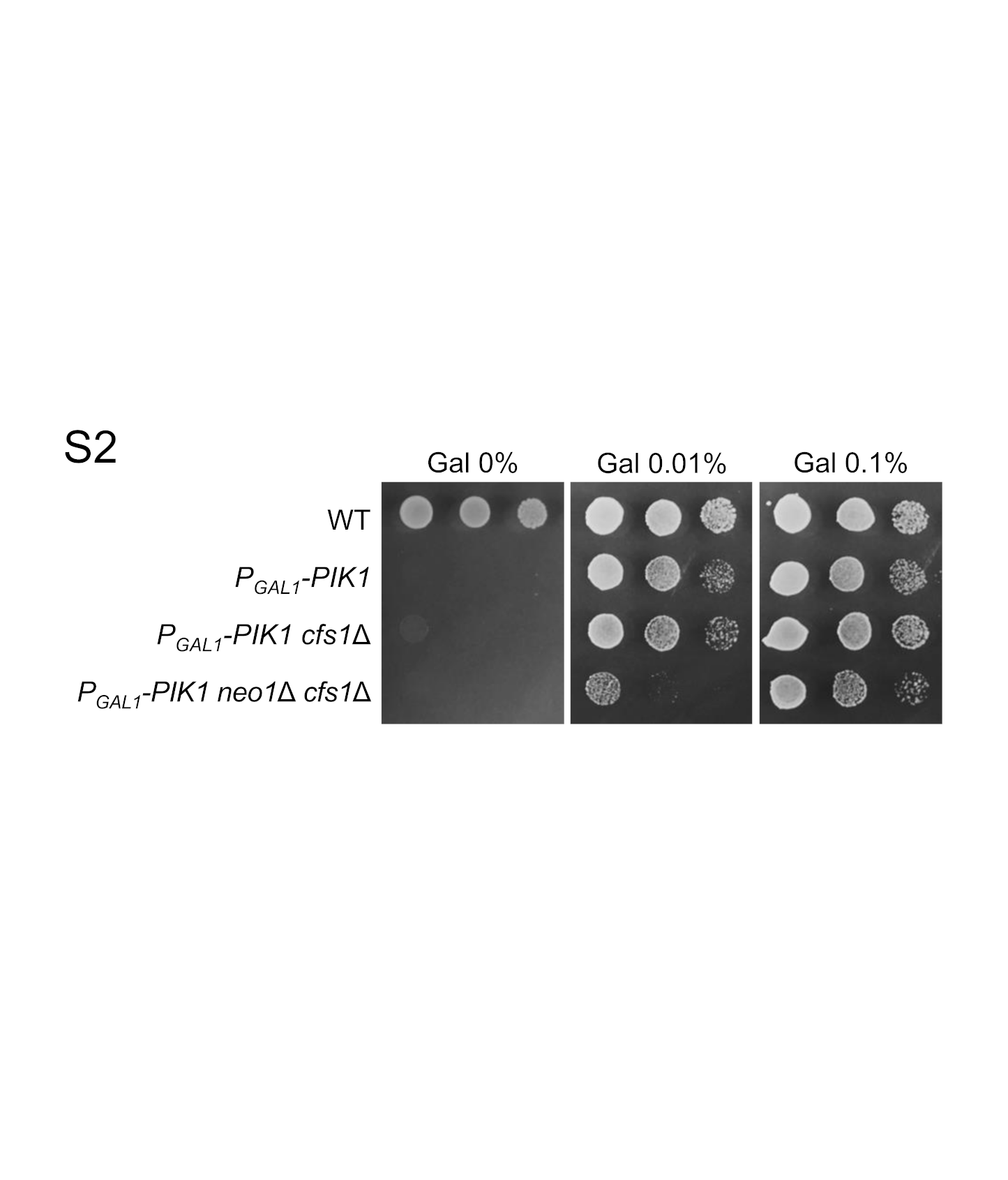

Supplement: S2 Fig — Cells were grown and spotted as in Fig 4A onto synthetic medium containing 2% glucose (Gal 0%) or 2% raffinose and 0.01% (Gal 0.01%) or 0.1% (Gal 0.1%) galactose, followed by incubation at 30°C for 2 d. The strains used were wild type (WT) (YKT38), PGAL1-PIK1 (YKT2219), PGAL1-PIK1 cfs1Δ (YKT2220), and PGAL1-PIK1 neo1Δ cfs1Δ (YKT2221). (TIF) [file pone.0236520.s002.tif]

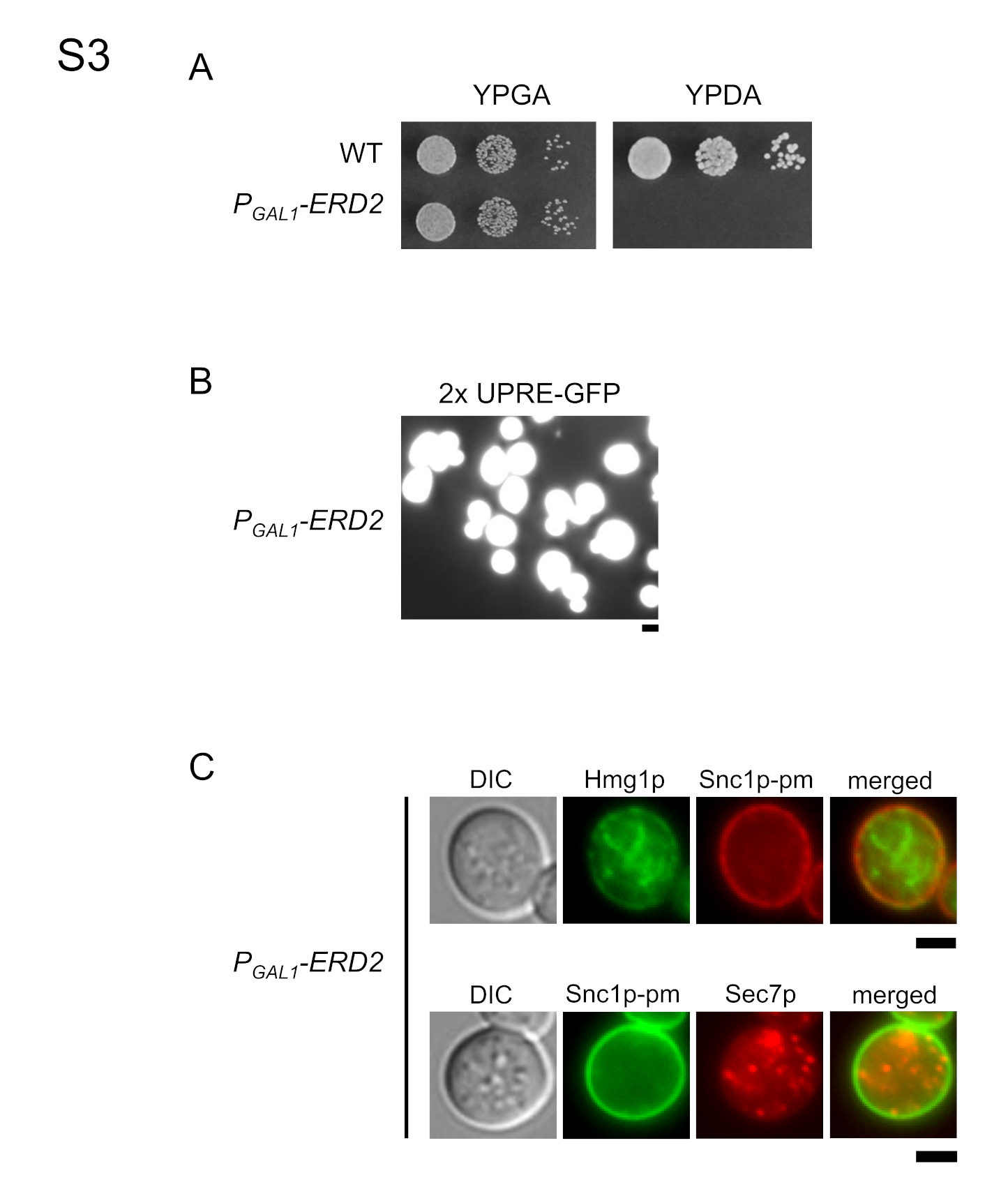

Supplement: S3 Fig — (A) The growth defect of the Erd2p-depleted mutant. Cells were grown and spotted as in Fig 1B onto YPGA (galactose) and YPDA (glucose) plates, followed by incubation at 30°C for 1.5 d. The strains used were wild type (WT) (YKT38) and PGAL1-ERD2 (YKT2222). (B) Induction of UPR in the Erd2p-depleted mutant. Yeast cells that express GFP under the control of 2× UPRE were cultured in YPDA as in Fig 1C. The strain used was the PGAL1-ERD2 (YKT2222), which carries 2× UPRE-GFP. (C) Localization of Hmg1p-GFP and Snc1-pm in the Erd2p-depleted mutant. Cells were cultured in YPDA as in Fig 1C. The strains used were the PGAL1-ERD2 with HMG1-GFP and mRFP1-SNC1-pm (YKT2223) or GFP-SNC1-pm and SEC7-mRFP1 (YKT2224). Bar, 5 μm. DIC, differential interference contrast. (TIF) [file pone.0236520.s003.tif]
