# Supplementary material for: A complex genetic interaction implicates that phospholipid asymmetry and phosphate homeostasis regulate Golgi functions
Source: PLoS One. 2020 Jul 30;15(7):e0236520. doi: 10.1371/journal.pone.0236520 (PMC7392219; doi:10.1371/journal.pone.0236520)
Supplement: S1 Table — (PDF) [file pone.0236520.s004.pdf]

S1 Table. *Saccharomyces cerevisiae* strains used in this study

Strain[a]		Genotype	Reference or source
YKT38	*MAT* **a**	*lys2-801 ura3-52 his3* Δ*-200 leu2* Δ*-1 trp1* Δ*-63*	[1]
YKT2085	*MAT* **a**	*TRP1::*P_{GAL1}*-NEO1 cfs1* Δ*::CaURA3*	[2]
YKT2134	*MAT* **α**	*KanMX6::*P_{GAL1}*-3HA-NEO1 TRP1*	This study
YKT2135	*MAT* **α**	*KanMX6::*P_{GAL1}*-3HA-NEO1 cfs1* Δ*::HIS3MX6 TRP1*	This study
YKT2136	*MAT* **a**	*KanMX6::*P_{GAL1}*-3HA-NEO1 cfs1* Δ*::HIS3MX6 erd1* Δ*::HphMX4 TRP1*	This study
YKT2137	*MAT* **α**	*cfs1* Δ*::HIS3MX6 erd1* Δ*::HphMX4*	This study
YKT2138	*MAT* **α**	*erd1* Δ*::KanMX6*	This study
YKT2139	*MAT* **a**	*LEU2::GFP-SNC1-pm*	This study
YKT2140	*MAT* **a**	*KanMX6::*P_{GAL1}*-NEO1 LEU2::GFP-SNC1-pm*	This study
YKT2141	*MAT* **a**	*KanMX6::*P_{GAL1}*-NEO1 cfs1* Δ*::HIS3MX6 LEU2::GFP-SNC1-pm*	This study
YKT2142	*MAT* **α**	*KanMX6::*P_{GAL1}*-NEO1 cfs1* Δ*::HIS3MX6 erd1* Δ*::HphMX4 LEU2::GFP-SNC1-pm*	This study
YKT2143	*MAT* **a**	*cfs1* Δ*::HIS3MX6 erd1* Δ*::HphMX4 URA3::GFP-SNC1-pm TRP1*	This study
YKT2144	*MAT* **a**	*erd1* Δ*::HphMX4 LEU2::GFP-SNC1-pm*	This study
YKT2145	*MAT* **a**	*PDR5-GFP::CaURA3*	This study
YKT2146	*MAT* **α**	*KanMX6::*P_{GAL1}*-3HA-NEO1 PDR5-GFP::CaURA3*	This study
YKT2147	*MAT* **α**	*KanMX6::*P_{GAL1}*-3HA-NEO1 cfs1* Δ*::HIS3MX6 PDR5-GFP::CaURA3*	This study
YKT2148	*MAT* **α**	*KanMX6::*P_{GAL1}*-3HA-NEO1 cfs1* Δ*::HIS3MX6 erd1* Δ*::HphMX4 TRP1*	This study
YKT2149	*MAT* **a**	*KanMX6::*P_{GAL1}*-3HA-NEO1 cfs1* Δ*::HIS3MX6 erd1* Δ*::HphMX4 PDR5-GFP::CaURA3 TRP1*	This study
YKT2150	*MAT* **a**	*HIS3MX6::*P_{GAL1}*-ERD1-GFP::KanMX6 MNN9-mRFP1::TRP1*	This study
YKT2151	*MAT* **a**	*HIS3MX6::*P_{GAL1}*-ERD1-GFP::KanMX6 SEC7-mRFP1::KanMX6*	This study
YKT2152	*MAT* **a**	*LEU2::GFP-SNC1-pm MNN9-mCherry::CaURA3 TRP1*	This study
YKT2153	*MAT* **a**	*KanMX6::*P_{GAL1}*-3HA-NEO1 LEU2::GFP-SNC1-pm MNN9-mCherry::CaURA3 TRP1*	This study
YKT2154	*MAT* **a**	*KanMX6::*P_{GAL1}*-3HA-NEO1 cfs1* Δ*::HIS3MX6 erd1* Δ*::HphMX4 LEU2::GFP-SNC1-pm MNN9-mCherry::CaURA3 TRP1*	This study
YKT2155	*MAT* **a**	*LEU2::GFP-SNC1-pm SEC7-mRFP1::CaURA3*	This study
YKT2156	*MAT* **a**	*KanMX6::*P_{GAL1}*-3HA-NEO1 LEU2::GFP-SNC1-pm SEC7-mRFP1::CaURA3*	This study
YKT2157	*MAT* **α**	*KanMX6::*P_{GAL1}*-3HA-NEO1 cfs1* Δ*::HIS3MX6 erd1* Δ*::HphMX4 LEU2::GFP-SNC1-pm SEC7-mRFP1::CaURA3*	This study
YKT2158	*MAT* **α**	*KanMX6::*P_{GAL1}*-3HA-NEO1 LEU2::GFP-SNC1-pm*	This study
YKT2159	*MAT* **α**	*URA3::OSH2-PH-GFP*	This study
YKT2160	*MAT* **α**	*KanMX6::*P_{GAL1}*-3HA-NEO1 URA3::OSH2-PH-GFP*	This study
YKT2161	*MAT* **α**	*KanMX6::*P_{GAL1}*-3HA-NEO1 cfs1* Δ*::HIS3MX6 URA3::OSH2-PH-GFP*	This study
YKT2162	*MAT* **α**	*KanMX6::*P_{GAL1}*-3HA-NEO1 cfs1* Δ*::HIS3MX6 erd1* Δ*::HphMX4 URA3::OSH2-PH-GFP*	This study
YKT2163	*MAT* **a**	*cfs1* Δ*::HIS3MX6 erd1* Δ*::HphMX4 URA3::OSH2-PH-GFP*	This study
YKT2164	*MAT* **a**	*erd1* Δ*::HphMX4 URA3::OSH2-PH-GFP*	This study
YKT2165	*MAT* **α**	*URA3::OSH2-PH-GFP SEC7-mRFP1::TRP1*	This study

YKT2166	*MAT* **α**	*KanMX6::P_{GAL1}-3HA-NEO1 URA3::OSH2-PH-GFP SEC7-mRFP1::TRP1*	This study
YKT2167	*MAT* **a**	*KanMX6::P_{GAL1}-3HA-NEO1 cfs1 Δ::HIS3MX6 URA3::OSH2-PH-GFP SEC7-mRFP1::TRP1*	This study
YKT2168	*MAT* **a**	*KanMX6::P_{GAL1}-3HA-NEO1 cfs1 Δ::HIS3MX6 erd1 Δ::HphMX4 URA3::OSH2-PH-GFP SEC7-mRFP1::TRP1*	This study
YKT2169	*MAT* **a**	*HIS3MX6::P_{PIK1}-GFP-PIK1 SEC7-mRFP1::KanMX6*	This study
YKT2170	*MAT* **a**	*TRP1::P_{GAL1}-NEO1 HIS3MX6::P_{PIK1}-GFP-PIK1 SEC7-mRFP1::KanMX6*	This study
YKT2171	*MAT* **a**	*TRP1::P_{GAL1}-NEO1 cfs1 Δ::CaURA3 HIS3MX6::P_{PIK1}-GFP-PIK1 SEC7-mRFP1::KanMX6*	This study
YKT2172	*MAT* **α**	*TRP1::P_{GAL1}-NEO1 cfs1 Δ::CaURA3 erd1 Δ::HphMX4 HIS3MX6::P_{PIK1}-GFP-PIK1 SEC7-mRFP1::KanMX6*	This study
YKT2173	*MAT* **a/α**	*neo1 Δ::HIS3MX6/NEO1 cfs1 Δ::HphMX4/cfs1Δ::HphMX4 ERD1/erd1 Δ::KanMX6 TRP1*	This study
YKT2174	*MAT* **a**	*KanMX6::P_{GAL1}-3HA-NEO1 cfs1 Δ::HIS3MX6 erd1 Δ::HphMX4 URA3::GFP-SNC1-pm TRP1*	This study
YKT2176	*MAT* **α**	*TRP1::P_{GAL1}-NEO1 cfs1 Δ::CaURA3 pho84 Δ::HIS3MX6*	This study
YKT2177	*MAT* **a**	*TRP1::P_{GAL1}-NEO1 cfs1 Δ::CaURA3 pho84 Δ::HIS3MX6 pho87 Δ::KanMX6 pho90 Δ::HphMX4*	This study
YKT2178	*MAT* **a**	*TRP1::P_{GAL1}-NEO1 cfs1 Δ::CaURA3 pho84 Δ::HIS3MX6 pho87 Δ::KanMX6 pho89 Δ::BleMX6 pho90 Δ::HphMX4*	This study
YKT2179	*MAT* **a**	*LEU2::mRFP1-SNC1-pm*	This study
YKT2180	*MAT* **α**	*HIS3MX6::P_{GAL1}-NEO1 LEU2::mRFP1-SNC1-pm*	This study
YKT2181	*MAT* **α**	*KanMX6::P_{GAL1}-3HA-NEO1 cfs1 Δ::HIS3MX6 LEU2::mRFP1-SNC1-pm TRP1*	This study
YKT2182	*MAT* **α**	*KanMX6::P_{GAL1}-3HA-NEO1 cfs1 Δ::HIS3MX6 erd1 Δ::HphMX4 LEU2::mRFP-SNC1-pm TRP1*	This study
YKT2183	*MAT* **a**	*URA3::HMG1-GFP*	This study
YKT2184	*MAT* **a**	*KanMX6::P_{GAL1}-3HA-NEO1 URA3::HMG1-GFP*	This study
YKT2185	*MAT* **a**	*KanMX6::P_{GAL1}-3HA-NEO1 cfs1 Δ::HIS3MX6 URA3::HMG1-GFP*	This study
YKT2186	*MAT* **α**	*KanMX6::P_{GAL1}-3HA-NEO1 cfs1 Δ::HIS3MX6 erd1 Δ::HphMX4 URA3::HMG1-GFP*	This study
YKT2187	*MAT* **α**	*cfs1 Δ::HIS3MX6 erd1 Δ::HphMX4 URA3::HMG1-GFP*	This study
YKT2188	*MAT* **a**	*erd1 Δ::HphMX4 URA3::HMG1-GFP*	This study
YKT2189	*MAT* **a**	*URA3::HMG1-GFP TRP1::NUP188-mRFP1*	This study
YKT2190	*MAT* **a**	*KanMX6::P_{GAL1}-3HA-NEO1 URA3::HMG1-GFP TRP1::NUP188-mRFP1*	This study
YKT2191	*MAT* **a**	*KanMX6::P_{GAL1}-3HA-NEO1 cfs1 Δ::HIS3MX6 URA3::HMG1-GFP TRP1::NUP188-mRFP1*	This study
YKT2192	*MAT* **α**	*KanMX6::P_{GAL1}-3HA-NEO1 cfs1 Δ::HIS3MX6 erd1 Δ::HphMX4 URA3::HMG1-GFP TRP1::NUP188-mRFP1*	This study
YKT2193	*MAT* **a**	*RTN1-GFP::KanMX6 SEC63-mRFP1::CaURA3*	This study
YKT2194	*MAT* **a**	*KanMX6::P_{GAL1}-3HA-NEO1 RTN1-GFP::KanMX6 SEC63-mRFP1::CaURA3*	This study
YKT2195	*MAT* **a**	*KanMX6::P_{GAL1}-3HA-NEO1 cfs1 Δ::HIS3MX6 RTN1-GFP::KanMX6 SEC63-mRFP1::CaURA3 TRP1*	This study
YKT2196	*MAT* **a**	*KanMX6::P_{GAL1}-3HA-NEO1 cfs1 Δ::HIS3MX6 erd1 Δ::HphMX4 RTN1-GFP::KanMX6 SEC63-mRFP1::CaURA3 TRP1*	This study
YKT2197	*MAT* **α**	*URA3::HMG1-GFP LEU2::mRFP1-SNC1-pm*	This study
YKT2198	*MAT* **α**	*KanMX6::P_{GAL1}-3HA-NEO1 URA3::HMG1-GFP LEU2::mRFP1-SNC1-pm*	This study
YKT2199	*MAT* **α**	*KanMX6::P_{GAL1}-3HA-NEO1 cfs1 Δ::HIS3MX6 erd1 Δ::HphMX4 URA3::HMG1-GFP LEU2::mRFP1-SNC1-pm*	This study
YKT2200	*MAT* **a**	*URA3::2x UPRE-GFP*	This study
YKT2201	*MAT* **α**	*KanMX6::P_{GAL1}-3HA-NEO1 URA3::2x UPRE-GFP TRP1*	This study
YKT2202	*MAT* **a**	*KanMX6::P_{GAL1}-3HA-NEO1 cfs1 Δ::HIS3MX6 URA3::2x UPRE-GFP TRP1*	This study

YKT2203	*MAT* **a**	*KanMX6::*P_{GAL1}*-3HA-NEO1 cfs1 Δ::HIS3MX6 erd1 Δ::HphMX4 URA3::2x UPRE-GFP TRP1*	This study
YKT2204	*MAT* **α**	*cfs1 Δ::HIS3MX6 erd1 Δ::HphMX4 URA3::2x UPRE-GFP TRP1*	This study
YKT2205	*MAT* **α**	*erd1 Δ::HphMX4 URA3::2x UPRE-GFP TRP1*	This study
YKT2206	*MAT* **a**	*HphMX6::*P_{GAL1}*-NEO1 cfs1 Δ::HIS3MX6 bst1 Δ::KanMX4*	This study
YKT2207	*MAT* **a**	*HphMX6::*P_{GAL1}*-NEO1 cfs1 Δ::HIS3MX6 emp24 Δ::KanMX4*	This study
YKT2208	*MAT* **a**	*HphMX6::*P_{GAL1}*-NEO1 cfs1 Δ::HIS3MX6 eps1 Δ::KanMX4*	This study
YKT2209	*MAT* **α**	*HphMX6::*P_{GAL1}*-NEO1 cfs1 Δ::HIS3MX6 erp1 Δ::KanMX4*	This study
YKT2210	*MAT* **α**	*HphMX6::*P_{GAL1}*-NEO1 cfs1 Δ::HIS3MX6 erp2 Δ::KanMX4*	This study
YKT2211	*MAT* **a**	*HphMX6::*P_{GAL1}*-NEO1 cfs1 Δ::HIS3MX6 rer1 Δ::KanMX4*	This study
YKT2212	*MAT* **a**	*TRP1::*P_{GAL1}*-NEO1 cfs1 Δ::CaURA3 van1 Δ::KanMX6*	This study
YKT2213	*MAT* **a**	*TRP1::*P_{GAL1}*-NEO1 cfs1 Δ::CaURA3 mnn10 Δ::KanMX6*	This study
YKT2214	*MAT* **a**	*TRP1::*P_{GAL1}*-NEO1 cfs1 Δ::CaURA3 mnn2 Δ::KanMX6*	This study
YKT2215	*MAT* **a**	*TRP1::*P_{GAL1}*-NEO1 cfs1 Δ::CaURA3 mnn5 Δ::KanMX6*	This study
YKT2216	*MAT* **a**	*TRP1::*P_{GAL1}*-NEO1 cfs1 Δ::CaURA3 mnn4 Δ::KanMX6*	This study
YKT2217	*MAT* **a**	*TRP1::*P_{GAL1}*-NEO1 cfs1 Δ::CaURA3 mnn6 Δ::KanMX6*	This study
YKT2218	*MAT* **a**	*TRP1::*P_{GAL1}*-NEO1 cfs1 Δ::CaURA3 mnn1 Δ::KanMX6*	This study
YKT2219	*MAT* **a**	*KanMX6::*P_{GAL1}*-3HA-PIK1*	This study
YKT2220	*MAT* **a**	*KanMX6::*P_{GAL1}*-3HA-PIK1 cfs1 Δ::HphMX4 TRP1*	This study
YKT2221	*MAT* **α**	*KanMX6::*P_{GAL1}*-3HA-PIK1 neo1 Δ::HIS3 cfs1 Δ::HphMX4 TRP1*	This study
YKT2222	*MAT* **a**	*KanMX6::*P_{GAL1}*-ERD2 URA3::2x UPRE-GFP TRP1*	This study
YKT2223	*MAT* **α**	*KanMX6::*P_{GAL1}*-ERD2 URA3::HMG1-GFP LEU2::mRFP1-SNC1-pm*	This study
YKT2224	*MAT* **a**	*KanMX6::*P_{GAL1}*-ERD2 SEC7-mRFP1::CaURA3 LEU2::mRFP1-SNC1-pm*	This study
YKT2225	*MAT* **a**	*KanMX6::*P_{GAL1}*-NEO1 erd1Δ::HphMX4 LEU2::GFP-SNC1-pm*	This study

[a]YKT strains are isogenic derivatives of YEF473 [3]. Only relevant genotypes are described.

[1] Misu K, Fujimura-Kamada K, Ueda T, Nakano A, Katoh H, Tanaka K. Cdc50p, a conserved endosomal membrane protein, controls polarized growth in *Saccharomyces cerevisiae* . *Mol Biol Cell* . 2003;14(2):730-47. doi: 10.1091/mbc.e02-06-0314. PubMed PMID: 12589066; PubMed Central PMCID: PMCPMC150004.

[2] Yamamoto T, Fujimura-Kamada K, Shioji E, Suzuki R, Tanaka K. Cfs1p, a Novel Membrane Protein in the PQ-Loop Family, Is Involved in Phospholipid Flippase Functions in Yeast. *G3 (Bethesda)* . 2017;7(1):179-92. Epub 2017/01/05. doi: 10.1534/g3.116.035238. PubMed PMID: 28057802; PubMed Central PMCID: PMCPMC5217107.

[3] Longtine MS, McKenzie A, Demarini DJ, Shah NG, Wach A, Brachat A, et al. Additional modules for versatile and economical PCR-based gene deletion and modification in *Saccharomyces cerevisiae* . *Yeast* . 1998;14(10):953-61. doi: 10.1002/(SICI)1097-0061(199807)14:10<953::AID-YEA293>3.0.CO;2-U. PubMed PMID: 9717241.
