# Supplementary material for: A complex genetic interaction implicates that phospholipid asymmetry and phosphate homeostasis regulate Golgi functions
Source: PLoS One. 2020 Jul 30;15(7):e0236520. doi: 10.1371/journal.pone.0236520 (PMC7392219; doi:10.1371/journal.pone.0236520)
Supplement: S2 Table — (PDF) [file pone.0236520.s005.pdf]

S2 Table. Plasmids used in this study

Plasmid	Characteristics	Reference or source
YEplac181	*LEU2* 2μm	[1]
pmCherry-Evc2-C2	*mCherry-evt-2 PH*	From Tomohiko Taguchi
pKT1444 [pRS416 GFP-SNC1-pm]	*P_{TPI1}-GFP-SNC1-pm URA3 CEN*	[2]
pKT1470 [YCplac33 NEO1]	*NEO1 URA3 CEN*	This study
pKT1476 [pRS416 GFP-SSO1]	*P_{TPI1}-GFP-SSO1 URA3 CEN*	[3]
pKT1487 [pRS416 GFP-PEP12]	*P_{TPI1}-GFP-PEP12 URA3 CEN*	[4]
pKT1491 [pRS315 GFP-SNC1-pm]	*P_{TPI1}-GFP-SNC1-pm LEU2 CEN*	[5]
pKT1755 [pRS416 mRFP1-Lact-C2]	*mRFP1-Lact-C2 URA3 CEN*	[5]
pKT2196 [pRS306 2x UPRE-GFP]	*2x UPRE-GFP URA3*	This study
pKT2197 [YEplac181 ERD1]	*ERD1 LEU2 2μm*	This study
pKT2198 [YEplac181-PHO87]	*PHO87 LEU2 2μm*	This study
pKT2199 [YEplac181 PHO90]	*PHO90 LEU2 2μm*	This study
pKT2200 [YEplac181 ERS1]	*ERS1 LEU2 2μm*	This study
pKT2201 [YEplac181 SAR1]	*SAR1 LEU2 2μm*	This study
pKT2202 [YEplac181 YIP1]	*YIP1 LEU2 2μm*	This study
pKT2203 [pRS416 P_{TPI1}-GFP-PHO87]	*P_{TPI1}-GFP-PHO87 URA3 CEN*	This study
pKT2204 [pRS416 P_{TPI1}-GFP-PHO90]	*P_{TPI1}-GFP-PHO90 URA3 CEN*	This study
pKT2205 [pRS316 mCherry-evt-2 PH]	*mCherry-evt-2 PH URA3*	This study

[1] Gietz RD, Sugino A. New yeast-Escherichia coli shuttle vectors constructed with in vitro mutagenized yeast genes lacking six-base pair restriction sites. *Gene.* 1988;74(2):527-34. doi: 10.1016/0378-1119(88)90185-0. PubMed PMID: 3073106.

[2] Lewis MJ, Nichols BJ, Prescianotto-Baschong C, Riezman H, Pelham HR. Specific retrieval of the exocytic SNARE Snc1p from early yeast endosomes. *Mol Biol Cell.* 2000;11(1):23-38. doi: 10.1091/mbc.11.1.23. PubMed PMID: 10637288; PubMed Central PMCID: PMCPMC14754.

[3] Reggiori F, Black MW, Pelham HR. Polar transmembrane domains target proteins to the interior of the yeast vacuole. *Mol Biol Cell.* 2000;11(11):3737-49. doi: 10.1091/mbc.11.11.3737. PubMed PMID: 11071903; PubMed Central PMCID: PMCPMC15033.

[4] Furuta N, Fujimura-Kamada K, Saito K, Yamamoto T, Tanaka K. Endocytic recycling in yeast is regulated by putative phospholipid translocases and the Ypt31p/32p-Rcy1p pathway. *Mol Biol Cell.* 2007;18(1):295-312. Epub 2006/11/08. doi: 10.1091/mbc.e06-05-0461. PubMed PMID: 17093059; PubMed Central PMCID: PMCPMC1751321.

[5] Mioka T, Fujimura-Kamada K, Tanaka K. Asymmetric distribution of phosphatidylserine is generated in the absence of phospholipid flippases in *Saccharomyces cerevisiae* . *Microbiologyopen.* 2014;3(5):803-21. Epub 2014/09/13. doi: 10.1002/mbo3.211. PubMed PMID: 25220349; PubMed Central PMCID: PMCPMC4234269.
