# Supplementary material for: A complex genetic interaction implicates that phospholipid asymmetry and phosphate homeostasis regulate Golgi functions
Source: PLoS One. 2020 Jul 30;15(7):e0236520. doi: 10.1371/journal.pone.0236520 (PMC7392219; doi:10.1371/journal.pone.0236520)
Supplement: Attachment — Submitted filename: Response to Reviewers.docx [file pone.0236520.s006.docx]

We thank the reviewers for valuable comments on our manuscript. We have thoroughly revised our manuscript according to the reviewers’ comments as described below.

Our responses to the comments of the Reviewer #1
*1) I have trouble grasping the meaning of some key statements in the manuscript. For instance, in the Abstract and on p. 4 (bottom) and p. 26 (middle), the authors state “our results suggest that flippase-mediated phospholipid redistribution is functionally vital not only in the cytoplasmic leaflet but also in the luminal leaflet of the Golgi membrane”. I do not see the added value of such a cryptic statement. The study also does not provide any concrete data on the lipid composition of the luminal leaflet in the Golgi of wildtype and mutant cells. I urge the authors to formulate a less ambiguous take-home message, one that focuses more on their principal experimental findings*.

We agree to the reviewer’s comments that our data do not lead us to any conclusion concerning a functional difference between the cytoplasmic leaflet and the luminal leaflet of the Golgi membrane. According to the reviewer’s suggestion, we have changed the sentences to “Our results suggest that phospholipid asymmetry and luminal Pi concentration coordinately regulate Golgi functions” in the Abstract (lines 32-33), the Introduction (lines 71-72), and the Discussion (lines 494-495).

*2) On p. 19 (bottom) and p. 20 (top), the authors state that their findings demonstrate that overexpression of Pho87p and Pho90p accelerates transport of luminally accumulated Pi to the cytoplasm, and conclude that elevated Pi levels in the Golgi lumen are responsible for the lethality of the Neo1p-depleted csf1/erd1 mutant cells. However, their experimental data provide only indirect evidence for this. Therefore, they should significantly tune down these claims, for instance by replacing the words “demonstrate” for “suggest” and “conclude” for “propose”.*

We agree to the reviewer’s comment that we should tone down our statement on the significance of elevated Pi levels in the Golgi lumen for the lethality of the Neo1p-depleted *cfs1 erd1* mutant. According to the reviewer’s suggestion, we have replaced the words “demonstrate” for “suggest” and “conclude” for “propose” (lines 362-365).

*3) To dissect the complex genetic interactions that implicate phospholipid asymmetry and phosphate homeostasis in regulating Golgi function, the authors analyzed the consequences of Neo1p depletion on Golgi function in wildtype, csf1 and csf1/erd1 mutant cells. As Erd1p has been suggested to transport Pi from the lumen of the Golgi, analyzing also the consequences of Neo1p depletion in csf1 mutant cells on Golgi functions would have been particularly informative. However, such experiments are not part of the present study. Why not?*

Although the reviewer suggests “analyzing the consequences of Neo1p depletion in *csf1* mutant”, we already analyzed this mutant in the original version. Judging from the context, we think that the reviewer suggests “analyzing the consequences of Neo1p depletion in *erd1* mutant”. However, because the Neo1p depletion in wild-type cells shows severe defects in Golgi functions, the Neo1p-depleted *erd1*Δ mutant would show similar severe defects unless the *erd1*Δ mutation suppresses the *neo1*Δ mutation. We actually constructed the Neo1p-depleted *erd1*Δ mutant expressing GFP-Snc1p-pm. This mutant exhibited massive accumulation of GFP-Snc1p-pm as the Neo1p-depleted and Neo1p-depleted *cfs1*Δ *erd1*Δ mutants. This result has been shown in Figure 1C in the revised version.

*4) p. 14 (top) “…the TGN is exposed to the accumulated PS” should read “…PS is accumulated in the cytosolic leaflet of the TGN”
5) p. 14 (end 1st para) “…the cytoplasmic leaflet of the TGN…. is exposed to PS” should be reformulated as indicated above.
6) Fig. 3 caption “The TGN… is exposed to PS and has no PI4P” should read “The cytoplasmic leaflet of the TGN … contains PS and has no PI4P”*

We thank the reviewer for pointing out our mistakes. We have corrected these points accordingly (lines 250, 254-255, 276, and 278).

Our responses to the comments of the Reviewer #2 *- In the abstract (lines 32-34) and in the discussion (lines 488-489), the authors propose that phospholipid redistribution is not only important in the cytosolic leaflet but also in the luminal leaflet. Do the authors mean that phospholipid asymmetry has an important impact not only on the cytosolic face of the membrane but also on the luminal one? I find the word ‘leaflet’ misleading in this case, as it restricts the impact of phospholipid asymmetry to the membrane itself*

This comment has also been made by the Reviewer #1. We agree to the reviewer’s comments that our data do not lead us to any conclusion concerning a functional difference between the cytoplasmic leaflet and the luminal leaflet of the Golgi membrane. According to the reviewer’s suggestion, we have changed the sentences to “Our results suggest that phospholipid asymmetry and luminal Pi concentration coordinately regulate Golgi functions” in the Abstract (lines 32-33), the Introduction (lines 71-72), and the Discussion (lines 494-495).

*- Introduction, line 47: spelling mistake ‘thorough’. But in fact, what do the authors mean by trafficking ‘through’ the membranes?*

We thank the reviewer for pointing out our mistake. As questioned by the reviewer, we do not mean any specific membrane trafficking pathway by “through”. Therefore, this word has been deleted. (line 47)

*- Introduction, lines 65-69. It is mentioned that ‘in the neo1Δcfs1Δ mutant, phospholipid asymmetry may not be normally regulated’. As cfs1 is a suppressor of neo1, it does not sound so obvious. Why should we envision defects in the neo1Δcfs1Δ mutant? Where does this assumption originate from? I think it would help the general reader to explain a bit better the rationale followed here. This is further substantiated by the fact that at lines 157-158 in the results section, it is claimed that the cfs1Δ mutation completely suppresses PE and PS exposure.*

As pointed out by the reviewer, our statement that “in the *neo1Δ cfs1Δ* mutant, phospholipid asymmetry may not be normally regulated” may not sound obvious. To make the sentence more consistent to readers, we have changed this sentence to “However, the *neo1*Δ *cfs1*Δ mutant may not be equivalent to the wild type in its Golgi functions, otherwise a set of these two genes including the essential *NEO1* gene would be dispensable.” (lines 65-67)
 *- Results, lines 182-184. The sentence is odd, as it starts by saying that Pdr5 accumulated in the Neo1-depleted cfs1Δerd1Δ mutant, and finishes with ‘although the accumulation of Pdr5-GFP was relatively low’. In addition, it’s not really clear form the figure that the accumulation is low.*

We thank the reviewer for these comments. To make how the accumulation of Pdr5-GFP was relatively low clearer, we have modified the sentence as follows. “…, although the accumulation of Pdr5p-GFP was relatively low compared to GFP-Snc1p-pm and GFP-Sso1p” (line 184). As to Fig 1D, the number of cells that showed clear accumulation of Pdr5p-GFP was lower compared to GFP-Snc1p-pm and GFP-Sso1p, but the cells that showed accumulation accumulated Pdr5p-GFP to an extent similar to those of GFP-Snc1p-pm and GFP-Sso1p. *- Results, lines 184-185. What is the evidence at this stage that the defect in protein trafficking is in the secretory rather than in the endocytic pathway? Especially given that Drs2/Cdc50 mutations interfere with endocytic retrieval of Snc1?*

Because we used GFP-Snc1p-pm, which is a mutant version that is not endocytosed, this protein does not enter the endocytic recycling pathway. However, it is known that Pdr5p is endocytosed and delivered to vacuoles. Thus, we have changed the sentence to “These results suggest that the Neo1p-depleted *cfs1*Δ *erd1*Δ mutant exhibits major defects in membrane trafficking pathways.” (lines 184-186) *- This is probably a very naive question, which is however relevant to several experiments displayed in the manuscript: in the legend to figure 1B, it is mentioned that cells are grown in YPDA, and then plated either on YPDA or YPGA; however, as Neo1 is an essential gene, how can cells grow with Neo1 under the control of a GAL promoter in a medium containing glucose as a carbon source?*

Because the catabolite repression is slow in the *GAL1* promoter, and because the *GAL1* promoter is a strong promoter, we need preincubation of the cells in YPDA medium to deplete Neo1p for ~12 h. Otherwise, the *PGAL1-NEO1* cells grown in YPGA continue to grow for ~12 h even in the YPDA medium. We have added “to deplete Neo1p” in the revised version (lines 193 and 199)

*- Legend to figure 1C, line 198. It is mentioned that ‘strains are cultured for 20h…’ In which medium are cells cultured?*

As written above, the cells were cultured in YPDA medium to deplete Neo1p. In the sentence pointed out by the reviewer, there was a redundant description that indicated that the cells were cultured more than 20 h in YPDA. This description, “and were cultured”, which is our mistake, has been deleted in the revised version (line 199). Because the repression of the *GAL1* promoter is not 100% in YPDA medium, some mutant requires depletion time of more than 12 hours. As shown in Fig 1B, the *PGAL1-NEO1 cfs1Δ erd1Δ* mutant showed some residual growth compared to the *PGAL1-NEO1* mutant, suggesting that the 12 h depletion is not enough to completely inhibit cell growth of the *PGAL1-NEO1 cfs1Δ erd1Δ* mutant. To observe the terminal phenotype of the *PGAL1-NEO1 cfs1Δ erd1Δ* mutant, we determined that twenty hours incubation in YPDA is appropriate for the *PGAL1-NEO1 cfs1Δ erd1Δ* mutant (line 199).  *- Results, line 248. ‘…the TGN is exposed to the accumulated PS’. The wording sounds odd to me if the aim is to say that PS is exposed to the cytosolic leaflet of the TGN. This wording can be found elsewhere in the manuscript. Moreover, how does PS distribute, using Lact-C2 and evt-2 probes, in Neo1-depleted cfs1Δerd1Δ mutant?*

These our mistakes were also pointed out by the reviewer #1, and all of them have been corrected appropriately to describe that PS is exposed to the cytoplasmic leaflet of the TGN (lines 250, 254-255, 276, and 278). As to the PS distribution using Lact-C2 and evt-2 probes, these probes were similarly colocalized with GFP-Snc1p-pm in the Neo1-depleted *cfs1*Δ *erd1*Δ mutant (Fig 3A and B). As shown in Fig 2C, GFP-Snc1p-pm was colocalized with Sec7p-mRFP1. These results suggest that PS is exposed to the cytoplasmic leaflet of the TGN in the Neo1-depleted *cfs1*Δ *erd1*Δ mutant (lines 254-255).  *- Results, line 264. It is suggested that absence of PI4P in the TGN may be the reason for membrane trafficking defects in the Neo1-depleted cfs1Δerd1Δ mutant. Isn’t it also true for the neo1Δ mutant? I suggest it’s worth recalling it so that a connection is made between the Neo1 flippase and PI4P.*

We thank the reviewer for pointing out that the Neo1p-depleted mutant should be included in the sentence. It is important to describe that the absence of PI4P may cause membrane trafficking defects in the Neo1p-depleted mutant. It has been added to the revised version (line 266).

*- Title of Fig 3. It is claimed that the TGN ‘has no PI4P’. I guess it would be fairer to say that PI4P levels dropped dramatically in the TGN, in the absence of sensitive assays to measure PI4P levels.*

We agree to the reviewer’s comment that we should not describe “has no PI4P”. According to the reviewer’s suggestion, we have changed it to “decreased level of PI4P” (line 277). *- In my opinion, the manuscript would gain in clarity if a simple schematic recalling the direction of phosphate transport envisioned for Erd1 and demonstrated for PHO transporters were added. It’s not immediately obvious that PHO transporters are involved in the uptake of phosphate and that would help understand the rationale of the experiments (lines 333-336).*

We thank the reviewer for the comment. According to the reviewer’s suggestion, we have added the schematic model as Fig 5A. In addition, we have also added Fig 5F to explain the other model, which is consistent with our data. *- Results, line 342. What is the phosphate concentration in a ‘normal’ SD medium (Fig 5B)?*

Phosphate concentration in SD medium is 7.3 mM according to the manufacturer. This has been added to Fig 5C and described in the legend in the revised version (lines 377 to 378). *- Results, lines 360-361. It’s probably a bit bold to claim that the authors demonstrated that ‘…Pho87 and Pho90 accelerated transport of the luminally accumulated Pi to the cytoplasm…’ in the absence of transport assays.*

This comment has also been made by the reviewer #1. According to the reviewers’ suggestion, we have replaced the word “demonstrate” for “suggest” (line 362). *- Legend to Fig 6A, line 409. Why the authors do not provide statistic tests here as for the other figures (figure 4 for instance)? Is the observed difference significant?*

According to the reviewer’s suggestion, we have performed statistic tests for Fig 6A (line 418) and Fig 1C (lines 204-205). Both tests gave a significant difference to the results as shown in the revised version. *- Results, lines 427-428. I’m not sure about the wording of the sentence. Is it the induction of UPR by DTT in the WT which is referred to? Why not comparing induction of UPR in the Neo1-depleted cfs1Δerd1Δ mutant with that of the WT in the absence of DTT? And the induction of UPR in the Neo1-depleted cfs1Δerd1Δ mutant does not seen higher than that of WT +DTT?*

As pointed out by the reviewer, to compare the induction of UPR in the Neo1-depleted *cfs1*Δ *erd1*Δ mutant with that of the WT in the absence of DTT, we have deleted the words “compared to that induced by DTT” in the revised version (line 436).
