# Supplementary material for: A complex genetic interaction implicates that phospholipid asymmetry and phosphate homeostasis regulate Golgi functions
Source: PLoS One. 2020 Jul 30;15(7):e0236520. doi: 10.1371/journal.pone.0236520 (PMC7392219; doi:10.1371/journal.pone.0236520)
Supplement: Attachment — Submitted filename: Response to Reviewers.docx [file pone.0236520.s007.docx]

Our responses to the comments of the Reviewer #1
*I urge the authors to have the manuscript proof-read by a native English speaker.*

The unrevised original version of our manuscript had already been checked by a leading company for English language editing. However, their work does not seem to be perfect, because the sentences pointed out by the reviewer #1 (e.g. comments 4, 5, and 6), which were also pointed out by the reviewer #2, were modified according to the editor’s suggestions. In the revised version, we finally made these sentences back almost to the original ones. We understand that this is because the editor would not be a specialist in our filed. Therefore, in the revised version, we checked the manuscript by ourselves for any kind of mistakes as much as possible.

Our responses to the comments of the Reviewer #2 *- However, I think it would help indicating the (perhaps putative) function of the genes which mutations are involved in ER retention (line 174), for instance in the legend to Figure S1A. The same holds for Fig S1B.*

According to the reviewer’s suggestion, gene (protein) functions have been included in S1 Fig A and B.

*- Line 205: '...fluorescent microscope' should read '...fluorescence microscope'?*

We thank the reviewer for pointing out our mistake. It has been corrected in the revised version (line 200).

*- Please indicate in the legend to Fig 5A what the rod-shaped grey drawing corresponds to.*

According the reviewer’s suggestion, we have indicated in the legend that the rod-shaped grey drawing corresponds to membrane proteins to be transported to the plasma membrane (line 371).
